# Applications and explorations of CRISPR/Cas9 in CAR T-cell therapy

**DOI:** 10.1093/bfgp/elz042

**Published:** 2020-01-17

**Authors:** Chenggong Li, Heng Mei, Yu Hu

**Keywords:** CAR T cells, CRISPR, Cas9, cancer immunotherapy, genome editing

## Abstract

Chimeric antigen receptor(CAR) T-cell therapy has shown remarkable effects and promising prospects in patients with refractory or relapsed malignancies, pending further progress in the next-generation CAR T cells with more optimized structure, enhanced efficacy and reduced toxicities. The clustered regulatory interspaced short palindromic repeat/CRISPR-associated protein 9 (CRISPR/Cas9) technology holds immense promise for advancing the field owing to its flexibility, simplicity, high efficiency and multiplexing in precise genome editing. Herein, we review the applications and explorations of CRISPR/Cas9 technology in constructing allogenic universal CAR T cells, disrupting inhibitory signaling to enhance potency and exploration of safer and more controllable novel CAR T cells.

**Key Points**

- CAR T-cell therapy has shown promising responses in both hematologic and solid cancers. However, there are some limitations awaiting for solutions, such as insufficient quantity and poor quality of autologous T cells, CAR T cell exhaustion and tumor suppressive microenvironments, potential self-killing and uncontrollable proliferation.
- Genomic editing technologies, especially CRISPR/Cas9 with flexibility, simplicity, high efficiency and multiplexing open a window to develop next-generation CAR T cells.
- CRISPR/Cas9 genomic editing technology holds promising explorations and applications to create next-generation CAR T cell products, including universal CAR T cells by disrupting endogenous TCR or HLA, more potent CAR T cells by ablating of inhibitory modulators and more controllable CAR T cells by adding inducible safe switches or suicide genes.
- CRISPR/Cas9 technology is unveiling a new era for CAR T cell therapy.

## Introduction

Cancer immunotherapy is the fourth mainstream treatment after surgery, chemotherapy and radiotherapy. Adoptive T-cell immunotherapy, particularly chimeric antigen receptor (CAR) T cell therapy, has revolutionized cancer therapy especially after the FDA approval of Kymriah and Yescarta (CD19-directed CAR T cells in B-cell leukemia and lymphoma) [1–3]. CARs are synthetic receptors typically containing an antibody-derived target-binding extracellular domain, a hinge region, a transmembrane domain and an intracellular signaling moiety capable of activating T cells [4,5]. T cells programmed with CARs can specifically recognize and kill antigen-expressing cells without the restriction of major histocompatibility complex (MHC). Clinical data has demonstrated that CAR T-cell therapy can induce durable complete remissions (CRs) in patients with a variety of hematologic and solid cancers, especially in relapsed/refractory acute lymphoblastic leukemia (ALL) and multiple myeloma with striking response rates of 80–100% [6–8]. Despite of promising efficacy of CAR T-cell therapy, there are several challenges awaiting for solutions, such as insufficient quantity and poor quality of autologous T cells, CAR T cell exhaustion and tumor suppressive microenvironments, potential self-killing and uncontrollable proliferation.

Optimization of the CAR T designs is supposed as one of the main tracks to tackle these limitations. The first generation of CAR T cells with only CD3 zeta intracellular chain was found to have modest proliferative and cytotoxic capacity [9–12]. The second generation of CARs contains a single costimulatory domain (CD28 or 4-1BB), proven to attain an improved efficacy and *in vivo* survival, whereas the third generation has two or more costimulatory domains (CD28, 4-1BB, ICOS or OX40), not superior to the second generation [13–15]. More functional elements are considered to be added to the next generation of CARs, like interleukins genes to increase potency, chemokine receptors genes to improve T-cell trafficking and on–off switches or suicide genes to enhance safety and controllability [16–18]. The structures and features of every generation of CAR-T are shown in Table 1.

**Table 1:** Structure and features of every generation of CAR-T

**Structure of CAR**		**First-generation CAR**	**Second-generation CAR**	**Third-generation CAR**	**Next-generation CAR**	**Universal CAR**
Similarities		An extracellular antigen-recognition region consisting of an scFv				
		A flexible hinge region derived from a CD8 molecule or CD28 or Fc region of an antibody				
		A transmembrane derived from CD8 or CD28				
Differences	Intracellular domain	only CD3ζ	CD3ζ	CD3ζ	CD3ζ	CD3ζ
			One costimulatory molecule: CD28 or 4-1BB	≥ 2 Costimulatory molecules: CD28, 4-1BB, ICOS or OX40	One costimulatory molecule: CD28 or 4-1BB	One costimulatory molecule: CD28 or 4-1BB
					Functional elements: interleukins, chemokine receptors, on-off-switches or suicide genes etc.	Knock-out of TCRs or/and HLAs
	*In vivo* Persistence	Low (days to 2 months)	Imporved (3 months to years)	Not superior to 2nd-generation	Exploration	Exploration
	Antitumor Effects	Low (ORR 0–40%)	Imporved (ORR depending on the tumor type)	Not superior to second-generation	Exploration	Exploration

The development of genomic editing technologies opens a window to accelerate the fourth generation of CAR T cells. There are currently three major genomic editing technologies, including zinc-finger nucleases (ZFNs), transcription activator-like effector nucleases (TALENs) and clustered regulatory interspaced short palindromic repeat/CRISPR-associated protein 9 (CRISPR/Cas9) [19–21]. Although ZFNs and TALENs have been applied to engineer T cells in clinical trials, the recognition of targetable DNA sequences is based on complicated protein conformation, a pair of Zn-finger binding domains or a pair of TALE DNA binding domains, accompanying with complex designs and relatively low gene-editing efficiencies [22,23]. CRISPR/Cas9, directed by a small guide RNA (sgRNA) to the target site, has become the most popular and developed of these tools due to its simplicity, flexibility, high efficiency and multiplexable genome editing capabilities [24–26]. A sgRNA-guided Cas9 nuclease induces a DNA double-stranded break at targeted genomic locations, subsequently repaired by non-homologous end joining (NHEJ) or homology-directed repair (HDR). NHEJ, an error prone repair pathway, can result in insertions or deletions of small nucleotide sequences and HDR can knock-in relatively large gene segments in the presence of a homology repair template at the site of interest [27–29]. Therefore, the combination with CRISPR/Cas9 technology will further expand the landscape of T-cell engineering. Besides knock-in of functional genes, such as interleukins and suicide genes, to product next-generation CAR T cells, other strategies comprises knock-out of endogenous genes, such as TCRs and MHCs, to develop ‘off-the-shelf’ universal CAR T cells [30], disruption of inhibitory receptors (such as PD-1 and TGF beta receptor) to ameliorate suppressive microenvironments [31,32], integration of the CAR cassette into the specific gene locus(such as TRAC and TET2) to improve efficiency and safety [33,34], deletions of target genes to avoid self-killing of CAR T cells [35]. CRISPR/Cas9 technology is unveiling a new era for CAR T-cell therapy. All gene-edited CAR T cells discussed here are shown in Table 2.

**Table 2:** Overview of the application of genomic editing technologies in CAR-T cells

**Target of CAR**	**Delivery of CAR**	**Target locus**	**Gene-editing method**	**Delivery**	**Editing efficiency**	**Reference**
CD19	SB electroporation	TRAC and TRBC	ZFNs	mRNA electrotransfer	15–37%	[33]
CD19	Lentivirus	TRAC and CD52	TALEN	mRNA electrotransfer	10–60%	[36]
CD19	AAV vector	TRAC (insert CAR to TRAC)	CRISPR/Cas9	Electroporation	~70%	[29]
CD19	SB electroporation	HLA-A	ZFNs	Nucleofection	40.70%	[40]
CD19	Lentivirus	B2M and TRAC	CRISPR/Cas9	RNA electroporation	52.55–65.21%	[26]
CD19		TRAC, B2M and PD-1			37.05–60.97%	
CD19	Lentivirus	B2M and TRAC	CRISPR/Cas9 (incorporating multiple gRNA cassettes in a single CAR vector)	Lentiviral vector	71.3 ± 6.7%	[42]
		TRAC, B2M and Fas			55.10%	
		TRAC, B2M and PD-1,CTLA-4			40.10%	
CD19	Lentivirus	TCR and B2M	CRISPR/Cas9	RNA electroporation	79.90%	[43]
		TRBC, B2M and PD-1			/	
PSMA	Lentivirus	dnTGF-βRII	/	Lentiviral vector	53.20%	[55]
CD19	Retrovirus	IL-15 and an suicide gene	Inducible caspase-9	Retroviral vector	65%	[57]
CD19	Retrovirus	Safety switch	Inducible caspase-9	Retroviral vector	61% ± 5%	[58]
CD19	Lentivirus	GM-CSF	CRISPR/Cas9	Lentiviral vector	82.20%	[61]
CD33	Lentivirus	CD33 in HSCs	CRISPR/Cas9	Electroporation	40–90%	[31, 64, 65]
CD7	Gammaretrovirus	CD7 in CAR T cells	CRISPR/Cas9	Electroporation	>80%	[66]

### Production of allogeneic universal CAR T cells

Although currently widespread-used autologous CAR T cells have shown promising results in cancer therapy, limitations exist. Almost 10–15% of enrolled patients were unable to receive infusions of CAR T cells because of poor quality and insufficient quantities of autologous T cells unavailable for manufacturing or rapid disease progression and even death before successful production of certain amount of CAR T cells [1–3]. A UPenn team recently reported a patient relapsing after infusion of anti-CD19 CAR T cells with CD19-negative leukemia that aberrantly expressed the anti-CD19 CAR because the CAR gene was unintentionally introduced into a single leukemic B cell during T-cell manufacturing [36]. The development of universal ‘off-the-shelf’ CAR T cells from healthy donors can circumvent the constraints and potentially be the mainstream direction in the future. The major barriers of such universal CAR T cell products are graft-versus-host disease (GVHD) and rejection of the infused allogeneic T cells. Endogenous αβ T cell receptors (TCRs) on adoptively transferred donor lymphocytes can recognize alloantigens in human leukocyte antigen (HLA) mismatched recipients resulting in GVHD; conversely, recognition of foreign HLA molecules on donor T cells may lead to rejection.

ZFNs and TALENs were successfully used to knock-out TCRα constant (TRAC) and TCRβ constant (TRBC) to generate TCR-negative CAR T cells to prevent GVHD without compromising CAR-mediated cytotoxicity [37,38]. Previous researches demonstrated that genetic knock-out of either *TRAC* or *TRBC* loci was sufficient to eliminate expression of αβTCR on the T cell surface [39]. The Cellectis firstly reported the generation of TALEN-edited allogeneic universal anti-CD19 CAR T(UCART19) cells in which *TRAC* and *CD52* genes were knocked out [40]. CD52 disruption in the CAR T cells allowed effective targeted depletion of patients’ autologous T cells using an anti-CD52 antibody (alemtuzumab). The first-in-man application of the products was two infants with high-risk CD19-positive ALL who achieved molecular remission after receiving the infusion of UCART19 cells and attained successful bridge-to-transplantation [41,42]. The remarkable results led to two clinical trials of UCART19 cells: CALM trial in adults and PALL trial in pediatric patients (NCT02746952 and NCT02808442). Pooled data of 20 patients showed acceptable and manageable safety with 15% (3/20) of severe cytokine release syndrome (CRS) and 10% (2/20) of G1 cutaneous acute GVHD as well as promising efficacy with 88% (14/16) of CR or CR with incomplete blood count recovery (CRi) and 86% (12/14) of minimal residual disease-negative [43]. A MSKCC group showed that directing a CD19-specific CAR to the TRAC locus using CRISPR/Cas9 technology not only minimized the risks of insertional oncogenesis and TCR-induced GVHD, but also enhanced T-cell potency and delayed T-cell exhaustion [33].

ZFNs were also used to target the HLA-A locus to permanently and completely eliminate HLA-I expression in primary and genetically modified human T cells used in clinical trials to evade rejection [44]. In addition, elimination of HLA heavy chains or beta-2-microglobulin (*B2M*), the non-polymorphic subunit of HLA-I complex, would prevent rapid rejection of allogeneic cells [45]. However, ideal universal CAR T cells should be silenced both TCR and HLA to avoid GVHD and rejection without reducing persistence and cytotoxicity *in vivo*. CRISPR/Cas9 has an obvious advantage in simultaneously multiplex and highly efficient genomic editing compared with ZFNs and TALENs. CRISPR/Cas9 was readily applicable to generate double-knock-out (*B2M* and *TRAC*, DKO) UCART19 cells with as similar safety and efficacy as wild-type anti-CD19 CAR T cells in preclinical studies [30]. One-shot CRISPR protocol for multiplex genome editing by incorporating multiple gRNA cassettes into a single CAR lentiviral vector was developed to generate DKO UCART19 cells [46]. By combining the lentiviral delivery of CAR with CRISPR RNA electroporation to co-introduce RNA encoding the Cas9 and gRNAs targeting endogenous TCR and B2M concurrently, an improved editing efficiency(~80%) was acquired to construct the DKO UCART19 cells that showed as potent antitumor activities as non–gene-edited CAR T cells both *in vitro* and in animal models [47]. However, the issue of whether such HLA-I negative CAR T cells will be the target of NK cells should be considered. Administering an anti-NK cell depletion antibody or engineering T cells with HLA-E are potential solutions to circumvent NK-mediated rejection [44, 48].

Recent advances in gene-editing technology, especially CRISPR/Cas9, allow for the production of universal CAR T cells starting from healthy donor in which the manufacture and quality of T cells can be preselected as well as GVHD and rejection can be avoided. The CRISPR/Cas9-modified universal CAR T cells need to be further tested for the safety and efficacy in clinical studies and there are currently six relevant ongoing clinical trials (Table 3).

**Table 3:** Current clinical trials about universal CAR T cells

**CAR target**	**Gene-editing technology**	**Locus of knock-out**	**Diseases**	**Phase**	**R&D Unit**	**Study location**	**NCT ID**
CD19	TALENs	TCR and CD52	B-ALL	1	Servier Group company	America, Europe	NCT02808442 NCT02735083 NCT02746952
CD19	CRISPR/Cas9	TRAC and HLA-I	ALL and NHL	1/2	Shanghai Bioray Laboratory Inc.	China	NCT03229876
CD19	CRISPR/Cas9	TRAC and B2M	B cell leukemia and lymphoma	1/2	Chinese PLA General Hospital	China	NCT03166878
CD19	CRISPR/Cas9	TCR and CD52	RR DLBCL	1	Nanjing Bioheng Biotech Co., Ltd	China	NCT04026100
CD123	TALENs	TCR and CD52	RR and Newly Diagnosed High-risk AML	1	Cellectis S.A.	Europe	NCT04106076 NCT03190278
CD123	TALENs	TCR and CD52	RR BPDCN	1	Cellectis S.A.	America	NCT03203369
BCMA	CRISPR/Cas9	TRAC and HLA-I	Multiple Myeloma	1/2	Shanghai Bioray Laboratory Inc.	China	NCT03752541

NHL, Non-Hodgkin lymphoma; RR, refractory or relapsed; DLBCL, diffuse large B-cell lymphoma; BPDCN, blastic plasmacytoid dendritic cell neoplasm.

### Disruption of inhibitory signaling molecules

The function of T cells was proven to play a significantly important role in the therapeutic effect of CAR T cells [49]. However, T cells are exposed to persistent antigen in patients with malignant tumors, resulting in T-cell exhaustion [50]. Exhausted T cells lose robust effector functions and express multiple inhibitory receptors, such as programmed cell death 1 (PD-1), cytotoxic T-lymphocyte antigen 4 (CTLA-4), domain-containing protein-3 (TIM-3) and lymphocyte-activated gene-3 (LAG-3), which inhibit T-cell proliferation and cytokine production leading to immune escape [51]. The inhibitory pathways also contribute to suppressive tumor microenvironment, a major barrier of CAR T-cell therapy in solid tumors. Immune checkpoint inhibitors, anti-PD-1/PD-L1 and anti-CTLA-4 antibodies, have shown promising clinical results and been approved by the FDA [52]. Thus, disruption of multiple inhibitory factors is expected to improve the potency of CAR T cells. Recent studies suggested that anti-CD19 CAR T cells with CRISPR-mediated triple-knock-out of the TRAC/TRBC, B2M and PD-1 genes displayed stronger antitumor functions in contrast to DKO UCART19 cells *in vitro* and in animal models [30,47]. High-fidelity Cas9s with the one-shot platform showed the feasibility of generating PD-1 and CTLA-4 dual inhibitory pathway-resistant DKO UCART19 cells by simultaneous disruption of quadruple genes [46].

The Fas receptor is a member of the tumor necrosis factor α (TNF-α) family of death receptors that mediate cell death [53]. Researches demonstrated that CAR T cell activity was attenuated due to cell Fas-FasL-dependent activation-induced cell death (AICD) [54]. Ren *et al*. [46] also utilized CRISPR/Cas9 technology to generate Fas-resistant universal CAR T cells that observed elevation of AICD resistance and prolonged survival.

Transforming growth factor-β (TGF-β) represses effector T-cell activities through binding the TGF-β receptors (TGFBRI and TGFBRII) to induce heterodimerization of the respective receptors and phosphorylation of the major TGF-β signal mediators SMAD2 and SMAD3, resulting in reduced cytokine production, cytotoxicity and amplification [55]. TGF-β also drives T-cell differentiation into regulatory T cells (Tregs) [56]. Thus, inhibiting TGF-β signaling, a potent immunosuppressive factor in a variety of solid tumors, has the potential to improve the immunosuppressive milieu. Previous studies demonstrated that TGF-β pathway could be blocked by using a dominant-negative TGFΒRII (dnTGF-βRII), which lacked the intracellular domain necessary for downstream signaling [57]. Foster *et al*. [31] used a clinical grade retrovirus vector to construct dnTGF-βRII-expressing human antigen-specific cytotoxic T lymphocytes (CTLs) and found that TGF-β-resistant CTLs had a functional advantage over unmodified CTLs in the TGF-β-secreting lymphoma [31]. The clinical trial (NCT00368082) showed that TGF-β-resistant CTLs could safely expand and persist in patients with Hodgkin lymphoma without lymphodepleting chemotherapy and induced complete responses [58]. It was testified that adding dnTGF-βRII to PSMA-targeted human CAR T cells promoted T-cell proliferation and augmented prostate cancer eradication [59]. Chang *et al*. recently described a novel TGF-β CAR containing a scFv based on the sequences of TGF-β-neutralizing antibodies, demonstrating the ability to not only inhibit endogenous TGF-β signaling but also convert TGF-β into a stimulant of T-cell growth [60]. Above results support the potential value of the countermeasure of using CRISPR/Cas9 technology to generate TGF-β-resistant CAR T or UCART cells to improve potency of engineering T cells in solid tumors.

### Exploration of safer and more controllable novel CAR T cells

Albeit unprecedented efficacy of CAR T-cell therapy, it is accompanied by serious and even life-threatening toxicities, including CRS, on-target/off-tumor toxicity, neurotoxicity, macrophage activation syndrome/ hemophagocytic lymphohistiocytosis and tumor lysis syndrome, which need to be paid more attention [61]. The most significant and common toxicity of CAR T-cell therapy is CRS, an inflammatory syndrome caused by multiple cytokines, including interferon γ, interleukin (IL)-1, IL-2, IL-4, IL-6, IL-8, IL-10, TNF-α and granulocyte/macrophage colony-stimulating factor (GM-CSF), produced by the CAR T cells themselves and by other cells [62]. Tocilizumab, IL-6 receptor blockade, was approved by FDA for treatment of CAR T cell-induced severe or life-threatening CRS [63]. Thus, blocking relevant cytokines signaling is a hopeful strategy to ameliorate the dilemma and CRISPR/Cas9 can effectively knock-out related molecules. Sterner *et al*. [64] described CRISPR/Cas9 mediated knock-out of GM-CSF and showed that GM-CSF-negative CAR T cells produced less GM-CSF without weakening antitumor activity *in vivo* compared to wild-type CAR T cells. Single or combined knock-out of other critical relevant cytokines in CAR T cells using CRISPR/Cas9 are needed to be further explored. Long-lasting B cell aplasia is a classical on-target/off-tumor toxicity of anti-CD19 and anti-CD20 CAR T-cell therapy [65, 66]. The insert of safety switches gene into CAR vector is a feasible method to terminate the effects without jeopardizing clinical responses. Diaconu *et al*. [67] demonstrated that the iC9 safety switch eliminated CD19-specific CAR T cells in a dose-dependent manner in a humanized mouse model, allowing either a selective containment of CAR T expansion in case of CRS or complete deletion on demand granting normal B-cell reconstitution.

There are two reported cases indicating the risks of unexpected situations in the manufacture of CAR T cells and potential carcinogenicity *in vivo*. One CD19-negative relapsed patient after CD19-targeted CAR T cell therapy was found that the CAR gene was unintentionally introduced into a single dominantly-proliferative leukemic B cell and its product bound in cis to the CD19 epitope on the surface of leukemic cells, masking it from recognition by CAR T cells [36]. Another case was a 78-year-old man with advanced relapsed/refractory chronic lymphocytic leukemia who obtained CR after the second infusion. Unexpectedly, 94% of CAR T cells at the peak of the response originated from a single clone in which lentiviral vector-mediated insertion of the CAR transgene disrupted the methylcytosine dioxygenase TET2 gene [34]. Therefore, there is a need to incorporate inducible safe switches or suicide genes into the CAR T cells, which can provide a means to eliminate the CAR T cells in case of unexpected toxicities. Hoyos *et al*. [68] generated a novel anti-CD19 CAR construct that incorporates the IL-15 gene and an inducible caspase-9(iC9)-based suicide gene and >95% of transgenic cells could be efficiently eliminated within 24 h upon pharmacologic activation of the suicide gene [68]. Adding inducible safe switches or suicide genes to generate more controllable and safer CAR T cells will be widespread-used by multiplexed CRISPR/Cas9 technology. There are three ongoing clinical trials (NCT02107963, NCT01822652 and NCT02439788) incorporating the iC9 construct into CAR T-cell products to provide a method to eliminate autologous CAR T cells in case of potential off-target toxicity.

### Other applications

CAR T-cells therapy has an obvious barrier in acute myeloid leukemia (AML) because myeloid-directed immunotherapy will eradicate normal as well as malignant cells, leading to bone marrow failure, as has been shown in several preclinical studies of CD33 or CD123 directed CAR T cell therapy [69, 70]. Several groups developed a novel approach to circumvent the problem with potent anti-CD33 CAR T cells followed by infusions of CRISPR/Cas9-modified CD33-konckout normal hematopoietic stem cells (HSCs), thus allowing persistent antigen-specific cytotoxicity along with reconstitution of effective hematopoiesis [35,71,72]. Extending the success of CAR T cells to T-cell malignancies is also problematic because most target antigens are expressed on both normal and malignant cells, resulting in CAR T-cell fratricide. CD7 is a transmembrane protein highly expressed in T-cell acute leukemia (T-ALL) and largely confined to T cells and natural killer cells. Studies showed that CD7-specific CAR T cell impaired expansion due to self-killing of the CAR T cells. Diogo *et al*. [73] explored that targeted disruption of the CD7 gene using CRISPR/Cas9 prior to CAR expression minimized fratricide in T cells and allowed the expansion of the CD7-knock-out anti-CD7 CAR T cells with robust antitumor activity for preclinical and potential clinical application. Hence, the CRISPR/Cas9 system can be applied to disrupt the targeted antigens to avoid self-killing of the CAR T cells and broaden the therapeutic index.

## Conclusion and outlooks

The unprecedented responses of CAR T cells in advanced malignancies promote the rapid growth of the therapeutic approach and the development of the smarter and commercialized CAR T cells is an inevitable mainstream trend, such as a split, universal and programmable CAR system to prevent relapse, mitigate overactivation and enhance specificity [74]. CRISPR/Cas9 genomic editing technology holds promising explorations and applications to create the next-generation CAR T-cell products, including universal CAR T cells by disrupting endogenous TCR and HLA, more potent CAR T cells by ablating inhibitory modulators, more controllable CAR T cells by adding inducible safe switches or suicide genes and novel CAR T cells by knock-out of the targeted antigens to avoid self-killing.

However, the gene-editing specificity and efficiency of CRISPR/Cas9 technology are of significant importance in therapeutic application. The first concern of CRISPR/Cas9 gene editing is off-target effects, which introduce random mutations, hence activating oncogenes or impacting tumor-suppressor genes to unintentional deleterious consequences [75]. Multiple strategies, such as careful selection of the target site, optimized sgRNA design and Cas9 activity, prior off-target detection assays, have been attempted to minimize the safe risks of off-target effects [76–78]. Attempts to increase HR frequencies using HR enhancers or NHEJ inhibitors are currently ongoing and may further promote precise gene engineering [79, 80]. Another challenge for therapeutic gene editing is efficient and nontoxic delivery into CAR T cells. There are three main methods to deliver CRISPR/Cas9 system, including a DNA plasmid-based system, an all-RNA-based system and a Cas9 ribonucleoprotein complex as delivery [81]. Viral vectors with high efficiency and potential hazards, such as mutagenesis, immunogenicity and off-target effects, are widely applied for donor DNA delivery and electroporation has emerged as new method to deliver CRISPR/Cas9 elements with safety, simplicity and flexibility [82]. Viral and non-viral vectors have specific merits and beneficial combinations of different delivery means are being explored to ensure efficiency and safety [33]. As technical progresses to reduce off-target effects and improve delivery efficiency, CRISPR/Cas9 technology provides an extraordinary potential to construct novel CAR T cells and streamlines the burgeoning realm of immunotherapy.

## Notes

**Chenggong Li** is a M.D. student working on universal CAR T cells using CRISPR/Cas9 genome-editing technology and safety and efficacy of novel CAR T cells in clinical trials.

**Heng Mei**, M.D. & PhD, is a professor and chief doctor with an expertise in CAR T-cell therapy on hematological malignancies and treatment of coagulation diseases.

**Yu Hu**, M.D. & PhD is a senior scientist and doctor focusing on diagnosis and therapy of hematological malignancies and fundamental researches of coagulation diseases.
